# Successful non-operative management of multiple intra-abdominal solid organ injury after blunt abdominal trauma: a case report

**DOI:** 10.11604/pamj.2022.43.54.35671

**Published:** 2022-10-03

**Authors:** Rajat Mahawar, Raju Shinde, Sangita Jogdand

**Affiliations:** 1 Department of General Surgery, Jawaharlal Nehru Medical College, Sawangi, Wardha, India; 2 Department of Pharmacology, Jawaharlal Nehru Medical College, Sawangi, Wardha, India

**Keywords:** Blunt trauma, non-operative management, multi-organ injuries, hemodynamic stability, case report

## Abstract

Injuries to the solid abdominal viscera are common after blunt trauma. The success of non-operative management of these injuries has led to recent extensions of this approach to managing higher-grade, more complicated injuries that are typically treated operatively. We reported a 19-year-old male who presented with abdominal pain and gross hematuria during the late hours due to a motor vehicle accident. Abdominal computed tomography scan revealed moderate hemoperitoneum, extensively devascularized spleen with laceration extending into the hilum, multiple tears in the left kidney extended to the hilum, and large perinephric hematoma suggestive of Grade V injuries (shattered spleen and left kidney). We managed the patient non-operatively until he improved and became ready for discharge from the hospital in stable good health status. In conclusion, this case brings to light a unique instance where severe grade multiple solid organ injury was successfully managed with a conservative approach.

## Introduction

Medical imaging and minimally invasive techniques advancements have greatly contributed to the expansion of Non-Operative Management (NOM) in more severe, complex, and even penetrating injuries. Non-operative management is now considered the gold standard of care in all hemodynamically stable injured adults without peritoneal signs, and numerous recent studies show success rates in excess of 80% [1,2]. Non-operative management of blunt splenic injury has a reported success rate of 95% or higher in pediatric patients and approximately 80% or higher in adults [1]. Non-operative management is also very successful in renal injuries with success rates of over 90% [2]. Notably, the success of the NOM depends on the solid organs involved, i.e., single or multiple organs injured, computed tomography (CT) scan diagnosis, and the patient's hemodynamic stability [3]. The contrast-enhanced CT scan is the imaging modality of choice in severely injured patients due to its high diagnostic accuracy in detecting injuries and accurately identifying the grade of injury [4]. Although NOM has a greater failure rate in multiple solid organ injuries, it is still the best therapeutic option in most hemodynamically stable patients [1,5]. We present a 19-year-old young male who presented with blunt abdominal trauma following a motor vehicle accident resulting in splenic and renal injuries grade V.

## Patient and observation

**Patient information:** a 19-year-old male presented to the emergency department with an alleged history of motor vehicle accidents due to slipping and falling from a two-wheeler during the late hours. The patient came with complaints of abdominal pain and gross hematuria. The patient revealed no signs of head injury or long bone injury, only bruises on the left flanks.

**Clinical findings:** on initial assessment, the patient was conscious and oriented, blood pressure was 98/60 mmHg, and tachycardia with a heart rate of 104 beats per minute. A thorough clinical examination of the abdomen only revealed mild tenderness in the left hypochondrium and no guarding or rigidity.

**Figure 1:**
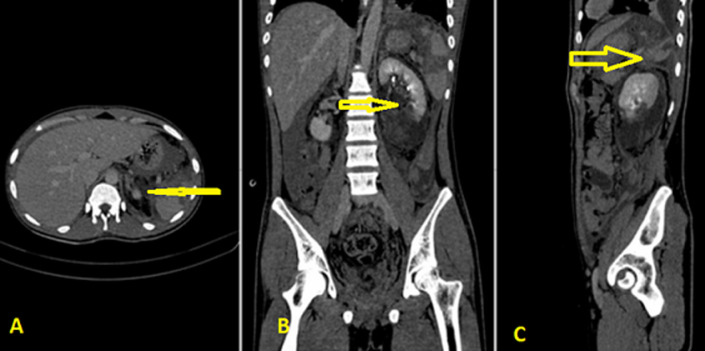
computed tomography scan showing lacerated and devascularized spleen and transected left kidney (arrows)

**Figure 2:**
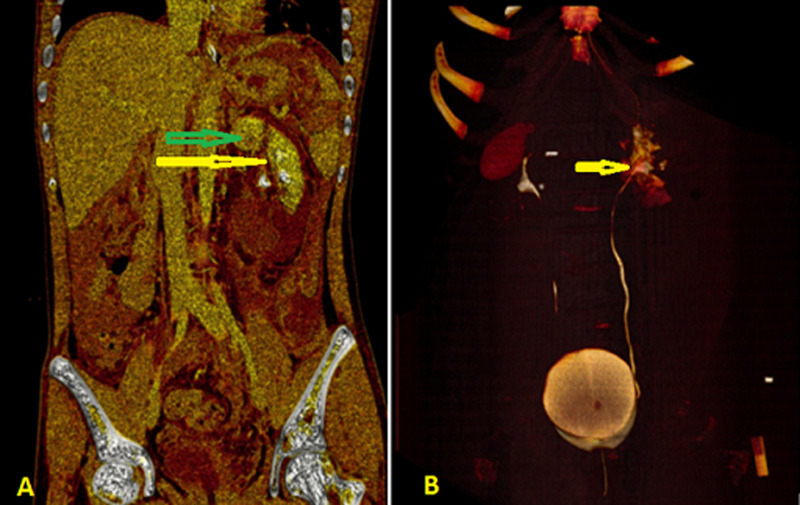
three (3-D) reconstructed computed tomography images showing the extent of injuries (arrows)

**Diagnostic assessment:** a focused assessment with sonography in trauma (FAST) revealed a fluid collection in the peri splenic region. Laboratory blood investigation revealed a hemoglobin of 8.7g/dL, white blood cell: 13600/mcL, platelets-200,000/ mcL, serum creatinine: 1.1 mg/dL, serum urea: 41 mg/dL. Urine analysis showed plenty of red blood cells and trace amounts of albumin. Simultaneous assessment and volume resuscitation were done with 1500 ml of isotonic crystalloid. After ascertaining hemodynamic stability, a decision to proceed with a CT scan of the abdomen was taken, which revealed devascularized spleen with laceration extending into the hilum (grade V), non-enhancing lower half of left kidney with laceration involving the upper, middle, and lower poles with extension into the hilum (grade V), and large perinephric hematoma (Figure 1). These findings suggest Grade V injuries (shattered spleen and left kidney) (Figure 2). The head and chest CT scans were normal.

**Therapeutic interventions:** since the patient was hemodynamically stable despite the progressive nature of the injury of both spleen and kidney, we faced a dilemma in deciding on lifesaving and ethical management for the patient. After a multidisciplinary discussion, a decision was taken to proceed with watchful NOM in surgical intensive care unit (ICU) blood transfusion with 1 unit of whole blood was done, and strict monitoring of the vital signs, fluid balance, antibiotic therapy, analgesics therapy, and daily blood work-up was paramount.

**Figure 3:**
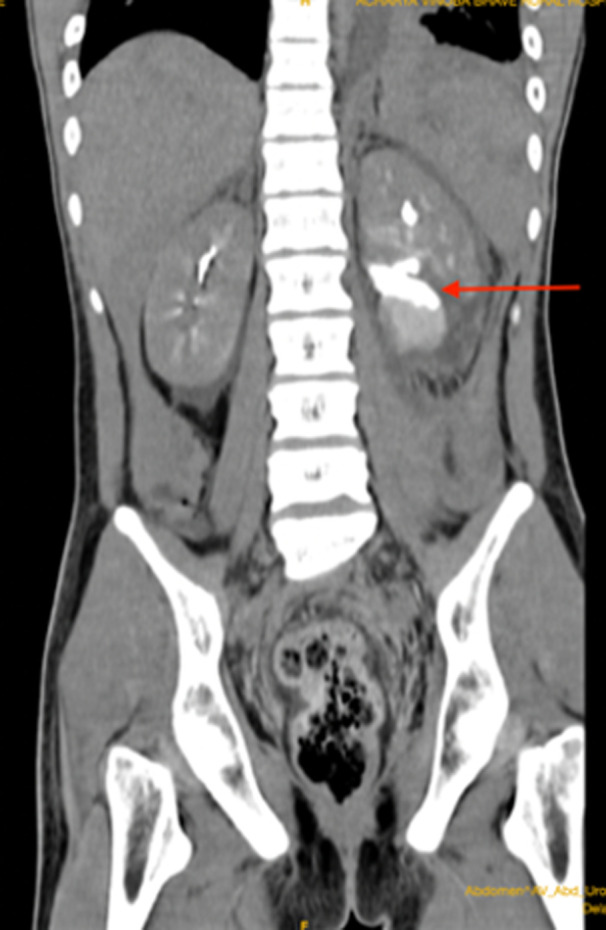
follow-up computed tomography scan showing contrast leak suggestive of urinoma (arrow)

**Follow-up and outcome:** a follow-up CT scan was done after 72 hours which revealed a large perinephric urinoma, approximately 246 ccs (Figure 3), for which a double j (DJ) stenting was inserted cystoscopically. Intravenous antibiotics (meropenem 1 gm thrice daily for ten days and Clindamycin 600 mg thrice daily for seven days) were instated during the hospital stay. Throughout the hospital stay, there was no worsening in the patient´s vitals or renal function. The rest of the blood picture remained unremarkable except for a marked (threefold) rise in the platelet count- upto 1,200,000/ mcL beginning from day 9, reaching to the maximum on day 13. The patient was discharged after 15^th^ days with an oral antibiotic (levofloxacin 500 mg once daily for ten days) and called for a subsequent follow-up after 15 days for a follow-up CT scan. The patient presented in good condition with no complications, and the follow-up CT scan showed a significant reduction in urinoma volume by approximately 120 ccs. For that, the DJ stent removal was done after 30 days. The patient was followed for 6 months, revealing excellent progress, with preservation of the booth spleen and kidney without secondary complications.

**Patient perspective:** I am glad readers worldwide will learn from my experience. I am very well-informed about the shared details and consent and approve of them. After reaching the hospital, a flurry of doctors and a battery of tests followed. Once the CT scan was done, the doctors explained the extent of the injury. We were explained about the ICU stay and possible operative procedure, which was kept on hold to avoid any unwarranted operative procedure while the treatment with Intravenous (IV) medications continued. Symptomatic relief also instilled confidence in the doctor's approach. The stay in the hospital was extended, but the doctors made sure they did not leave any aspect unattended. I am glad and thankful to have recovered without undergoing a major operative procedure.

**Informed consent:** written informed consent was obtained from the patient for participation in our study.

## Discussion

Surgery is once the treatment of choice for individuals with multiple solid organ injuries. Nevertheless, NOM has steadily replaced surgery as the line of management throughout the last two decades [1]. This shift in NOM has been aided by advancements in critical care monitoring and CT scan, as well as increased use of interventional radiology procedures, such as angioembolization, percutaneous drainage, and nephrostomies insertion [6]. However, despite developing relatively less invasive procedures, higher grades of solid organ injuries still need to be treated surgically if the hemodynamic stability is compromised [7]. So far number of retrospective studies regarding NOM have been carried out, but only a few prospective talks about multiple organ injuries and their outcome [1]. A few cases have been reported with multiple organ injuries treated successfully with NOMs, such as Georgios *et al*. and Soma *et al*. [4,6]. The organ injury scaling (OIS) system was developed by American association for the surgery and based on the magnitude of anatomic disruption and is graded as 1 (minimal), 2 (mild), 3 (moderate), 4 (severe), 5 (massive), and 6 (lethal) [8]. Low grades of solid organ injury (I-III) as a dictum are managed with NOM. High-grade damage, extensive hemoperitoneum, active bleeding symptoms on CT scan, older patient age, and multiple solid organ injury are all risk factors for NOM failure [8,9]. The present case suffered Grade V injuries (shattered spleen and left kidney) and was successfully managed with NOM.

In the case of multiple advanced solid organ injuries, if the patient is hemodynamically stable and facilities for strict monitoring with a highly specialized trauma center are available, NOM may be considered [1]. Compared to injury to a single organ, NOM in hemodynamically stable patients with multiple intra-abdominal organ injuries is associated with longer intensive care unit, hospital stays, comorbidities, and, more commonly, NOM failure [4,10]. The failure rate of NOM in isolated solid organ injury and multiple solid organ injuries were 11% to 40 and higher than 70%, respectively [11]. Blunt injury to the spleen does not cause hemodynamic instability, peritonitis, or other abdominal injuries that require surgery, NOM is the standard treatment [12]. The failure rate of NOM increases with the increasing grades of injury, from 4.8% in grade I to 75% in grade V [13]. In blunt splenic trauma, the risk of delayed bleeding is as high as 20% in grade III injuries, 50% in individuals with active contrast extravasation, and 70% in patients with extensive hemoperitoneum [12]. Non-operative management in the case of blunt renal injury was limited to low-grade injuries (grades I-III) with stable hemodynamics, but now it is gaining traction in selective high-grade injuries as well. It was reported that, in cases of grade IV renal injury, NOM is not always enough, with 11% of patients requiring renal exploratory surgery, 25% requiring embolization, and 27% requiring ureteral stenting, as was done in our patient. The consensus is that NOM is absolutely contraindicated in cases of renal vascular pedicle avulsion, life-threatening bleeding, and the persistence of a pulsatile and increasing hematoma [14].

The ureteric stent should be kept in place for at least three weeks or until radiographic proof of full resolution is obtained. In situations of expanding urinomas, tiny urinomas with clinical sepsis increased discomfort, ileus, and fistula development, percutaneous drainage should be considered. However, in cases of proximal collecting system avulsion (renal pelvis or proximal ureter) with large medial urinoma or gross contrast leakage in the pyelographic phase combined with the non-opacified distal part of the ureter and where the patient has failed minimally invasive urine leakage control techniques, operative intervention is usually required [12]. There is still no agreement on how long is adequate and when it is safe to discharge from the hospital. Most NOM patients (95%) failed during the first 72 hours of admission, indicating that patients should be monitored for at least 3-5 days in the hospital [15]. Our patient was discharge in 15 days with good condition. The patient was followed for 6 months, revealing an excellent progress, with preservation of the booth spleen and kidney without secondary complications. A similar case with similar result was reported by Laculiceanu *et al*. [16].

## Conclusion

The presence of multiple solid organ injuries should not preclude the use of non-operative management. Although high grades of solid organ injury traditionally entail operative intervention. This case highlights the efficacy of NOM in a polytrauma patient. We managed combined grade V injuries of the spleen and kidney conservatively, and the patient was discharged without complications. Computer tomography scan proved to be critical in the management. Non-operative management should only be appropriate for highly selected patients in conjunction with good clinical assessment by an experienced surgical team in the setting of a highly specialized trauma center.
